# Novel nomograms for survival and progression in HPV+ and HPV- oropharyngeal cancer: a population-based study of 1,542 consecutive patients

**DOI:** 10.18632/oncotarget.12335

**Published:** 2016-09-29

**Authors:** Christian Grønhøj Larsen, David H. Jensen, Amanda-Louise Fenger Carlander, Katalin Kiss, Luise Andersen, Caroline Holkmann Olsen, Elo Andersen, Emilie Garnæs, Finn Cilius, Lena Specht, Christian von Buchwald

**Affiliations:** ^1^ Department of Otorhinolaryngology, Head and Neck Surgery and Audiology, Rigshospitalet, University of Copenhagen, Copenhagen, Denmark; ^2^ Department of Pathology, Rigshospitalet, University of Copenhagen, Copenhagen, Denmark; ^3^ Department of Oncology, Herlev Hospital, University of Copenhagen, Copenhagen, Denmark; ^4^ Centre for Genomic Medicine, Rigshospitalet, University of Copenhagen, Copenhagen, Denmark; ^5^ Department of Oncology, Rigshospitalet, University of Copenhagen, Copenhagen, Denmark; ^6^ Department of Pathology, Roskilde Hospital, Roskilde, Denmark

**Keywords:** oropharyngeal cancer, human papillomavirus, survival, nomogram

## Abstract

**Background:**

No study has combined tumour and clinical covariates for survival to construct an individual risk-profile for overall survival (OS), time to progression (TTP), and survival after progression (SAP) in patients with HPV+ and HPV– oropharyngeal squamous cell carcinoma (OPSCC). Based on the largest-to-date, unselected, population-based cohort of patients diagnosed with OPSCC, we performed a comprehensive analysis of long-term OS, TTP, and SAP and constructed novel nomograms to evaluate patients' prognoses.

**Results:**

At a median follow-up of 4.0 years (range: 0.8–15.8 yrs.), 690 deaths were recorded. The 5-year OS, TTP, and SAP for the HPV+/p16+ subgroup were 77%, 82%, and 33, vs. 30%, 66%, and 6% for the HPV–/p16– group (*P* < 0.01). 376 patients failed to maintain disease control with a median TTP of 13 months in the HPV+/p16+ subgroup vs. 8.5 months in the HPV–/p16– subgroup (*P* < 0.05). HPV combined with p16 status remained one of the most informative covariates in the final Cox regression model for OS, TTP, and SAP.

**Methods:**

We included all patients diagnosed with OPSCC (*n* = 1,542) between 2000–2014 in Eastern Denmark. Survival rates were estimated by the Kaplan-Meier method. A multivariate Cox regression model was used to construct predictive, internally validated nomograms.

**Conclusion:**

The HPV+/p16+ subgroup had improved OS, TTP, and SAP compared with other combinations of HPV and p16 after adjusting for covariates. Nomograms were constructed for 1-, 5- and 10-year survival probability. Models may aid patients and clinicians in their clinical decision making as well as in counselling, research, and trial design.

## INTRODUCTION

In the Western world, the main risk factor for developing oropharyngeal squamous cell carcinoma (OPSCC) is infection with high-risk human papillomavirus (HPV); while, a smaller proportion is related to the high consumption of alcohol and smoking tobacco. Patients with HPV-associated OPSCCs have improved survival, which may be related to a different mutational profile [1, 2], histopathology [3], and clinical features [4]. The first reports of improved survival for HPV-positive (HPV+) OPSCCs were published in the early 2000's [5, 6]. Shortly after, p16 (a surrogate-marker for HPV- infection) was demonstrated to have prognostic value [7, 8]. The importance of HPV and p16 has since been confirmed, and the combination (e.g. HPV/p16-status) has shown better prognostication [9]. However, long-term results are missing for overall survival (OS), time to progression (TTP), and survival after progression (SAP), especially their relationship to clinico-pathological characteristics from a large, population-based cohort.

Progression is a strong predictor of survival in patients with OPSCCs. The subgroup of patients who are HPV and p16-positive have a lower risk of recurrence and improved SAP compared with patients without HPV/p16 [10–12]. These findings are based on selected patients from small cohort-studies examining only p16 or HPV without a detailed account of progression. Therefore, HPV+ OPSCC may have an increased OS due to a better response to salvage therapy (i.e. SAP). A nomogram is a graphic demonstration of a statistical model for calculating the cumulative effect of weighted variables on the probability of a particular outcome, and enables continuous estimation of the probability of specific outcomes (i.e. death or progression). Furthermore, they can combine multiple independent variables while considering the prognostic weight for each variable when calculating the probability of an outcome. Although nomograms have been developed to predict clinical end-points for patients with several types of malignancies, to the best of our knowledge, this is the first study addressing OS, TTP, and SAP in OPSCCs [13–17].

To avoid selection-bias, we aimed to include all patients diagnosed with OPSCC in the Eastern part of Denmark, which has an approximate population size of 2.4 million inhabitants. The cohort is well described and includes detailed information on both tumour (HPV, p16, stage, site) as well as clinical characteristics (follow-up, treatment, progression) with virtually no patients lost to follow-up. The primary aim was to identify tumour- and patient-factors associated with OS, TTP, and SAP. Due to our unique, complete population-based cohort spanning 15-years, we were able to construct and validate reliable, predictive nomograms.

## RESULTS

### Population demographics

**Figure 1:**
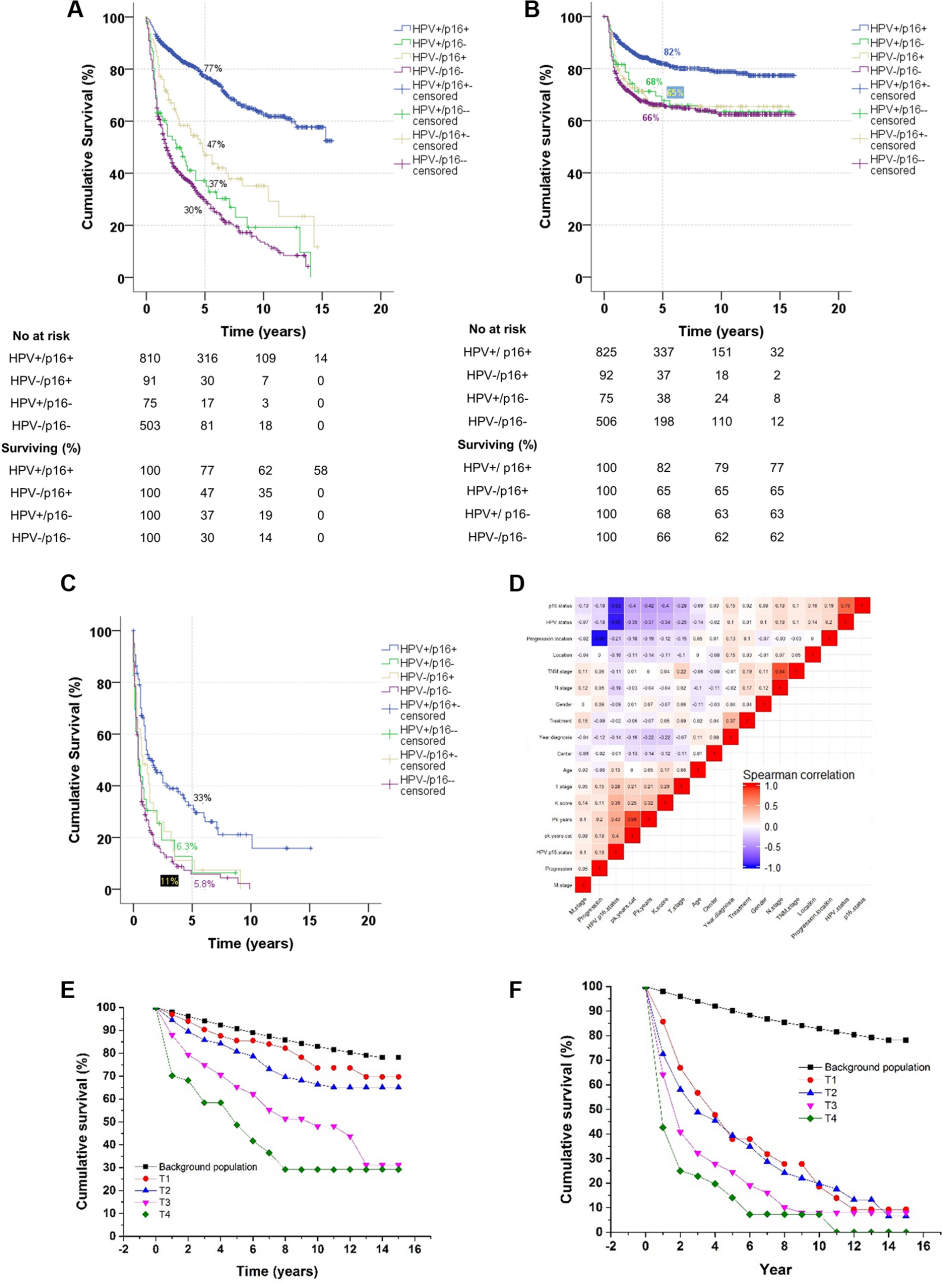
Kaplan-Meier curves for (**A**) overall survival, (**B**) time to progression, and (**C**) survival after progression. Correlation heatmap between variables (**D**). Comparison between survival in an age- and gender-matched background population and the HPV+/p16+ subgroup (**E**) and the HPV−/p16− subgroup (**F**) based on T classification of tumours.

A total of 1,542 patients diagnosed with OPSCC between 2000–2014 were included, and their characteristics are summarized in Table 1 with univariate hazard ratios for OS. The majority of patients were males (72%), with a median age of 60 years at diagnosis, and typically presented with tumours in an advanced nodal stage, but with small primary tumours (Table 1). Of the total cohort, 54% were stage III or IV, 61% had T1 or T2 primary tumours, and 60% had N2 or N3 nodal classification (Table 1). The median follow-up for patients alive at the last date of follow-up was 4.0 years (range, 0.8 to 15.8 years). Tobacco use was high with a median number of pack-years of 27 (mean 30). Subsequently, we examined which of the variables were correlated (Figure 1D). It was observed that the subgroup of patients with an HPV+/p16+ tumour correlated with: a shorter smoking history, higher N-stage, better performance status, lower T-stage, and younger age (Figure 1D).

**Table 1:**
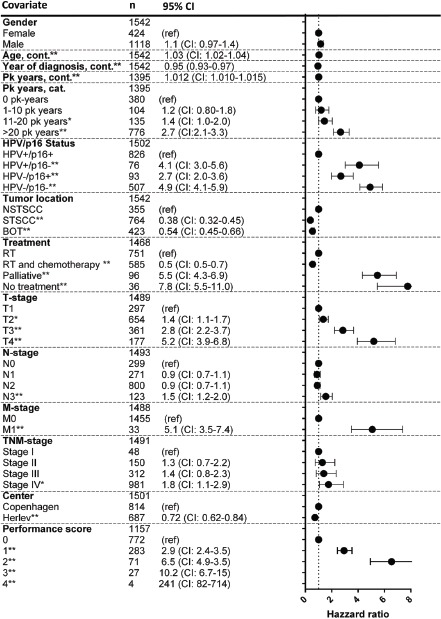
Patient characteristics and their relationship to overall survival

Footnote: The hazard ratios for age, year of diagnosis, and pack years represents the hazard ratio per year increase. RT: radiotherapy. BSCC: Base of tongue squamous cell carcinoma. STSCC and NSTSCC: Specified- and non-specified tonsillar squamous cell carcinomas.

### Univariate and multivariate analysis of factors influencing overall survival

In the OS analysis, 1,524 patients (18 were lost to follow-up) were included, with a total of 690 deaths. In the total cohort, 1,336 patients were treated with curative regimes; of these patients, 543 died during follow-up. The subgroup of HPV+/p16+ had a markedly better prognosis compared with the other combinations of HPV/p16 (Table 1 and Figure 1A) (*p* < 0.05). Other factors significantly associated with a poorer OS in the univariate analysis were: pack years of smoking, treatment, tumour location, and T, N, and M classification (Table 1). We subsequently constructed a multivariate Cox regression model to examine which factors independently predicted OS. The HPV/p16 status was an independent predictor for OS, even when adjusted for T-stage, N-stage, treatment, smoking history, age, and performance status (Supplementary Table S1). To better illustrate any excess mortality in the HPV+/p16+ and HPV−/p16− subgroups, Kaplan-Meier survival curves were compared with the mortality rates of the background population matched for age, gender, and calendar period (Figure 1E and 1F). Interestingly, patient with stage T1-T2 tumours in the HPV+/p16+ subgroup had almost no excess mortality compared with the background population (Figure 1E), which was in stark contrast to the HPV−/p16− subgroup (Figure 1F).

### Univariate and multivariate analysis of factors influencing time to progression

In total, 376 patients experienced a progression, with 153 (19%) in the HPV+/p16+ subgroup vs. 182 (36%) in the HPV−/p16− subgroup (*P* < 0.01). The subgroup of HPV+/p16+ had a significantly longer TTP compared with the other combinations of HPV/p16 (Figure 1B) (*P* < 0.05). The median TTP for the total cohort was 9.7 months. The median TTP was 13 months for the HPV+/p16+ subgroup and 8.5 months (*P* < 0.05) for the HPV−/p16− subgroup. Median time to loco-regional progression was 7.6 months for the HPV+/p16 subgroup and 7.4 months for the HPV−/p16− group. The corresponding figures for distant progression were 18 and 11 months, respectively, (*P* < 0.05). The univariate analysis of factors influencing TTP included: pack years of smoking, HPV/16 status, and T, N, and M classification of tumours (Supplementary Table S2). A multivariate Cox regression model identified HPV/p16 status as an independent predictor of TTP with a hazard ratio of 3.0 (95% CI: 2.3–3.8) in the HPV−/p16− subgroup compared with the HPV+/p16+ subgroup, after adjustment (Supplementary Table S3).

### Univariate and multivariate analysis of factors influencing survival after progression

The median SAP was 13 months in the HPV+/p16+ subgroup vs. 6 months in the HPV−/p16− subgroup (*P* < 0.05). The HPV+/p16+ status also independently influenced SAP positively with a hazard ratio of 2.2 (95% CI: 1.6–2.9) compared with the HPV–/p16– subgroup (Supplementary Table S4), when adjusted for other significant covariates. Performance status and progression location also independently influenced SAP (Supplementary Table S4).

### Predictive nomograms

**Figure 2:**
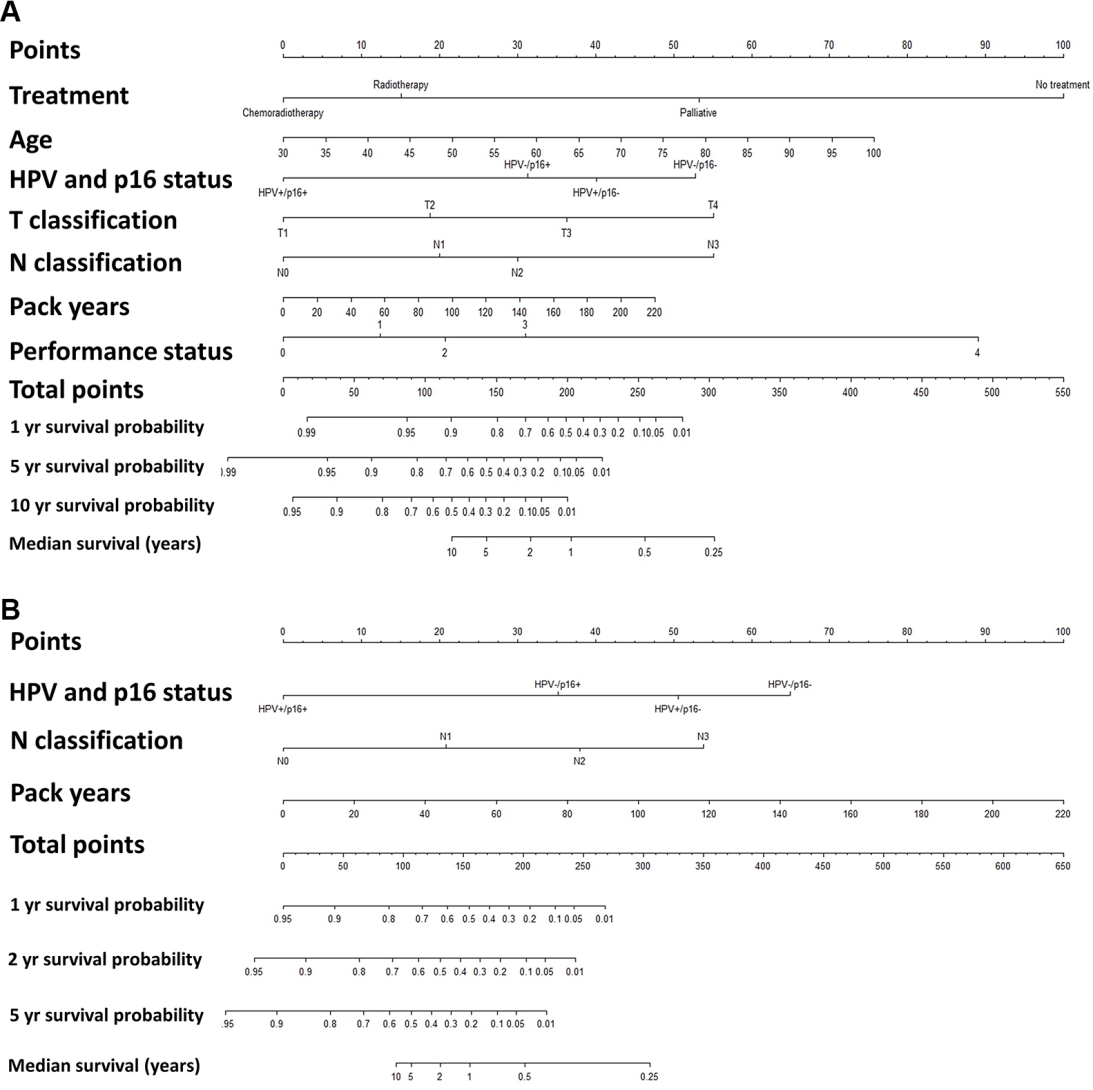
Nomogram for overall survival (A) and time to progression (B) The nomogram is used by totalling the points identified on the top scale for each independent covariate. The total points scale is used to identify the estimated median survival time (years) and the probability of 1-, 5-, and 10-year survival.

Nomograms (Figure 2A and 2B) were constructed to predict survival using the independent covariates identified in the multivariate Cox regression models. The median total points for the 1,523 patients used to fit the multivariate Cox model for OS was 101 (range, 0–421). The nomogram is used by totalling the points identified on the top scale for each independent covariate which is identified on the total points scale to identify the estimated median survival time (years) and the probability of 1-, 5- and 10-year survival.

The bias-corrected concordance index for this nomogram was 0.79 based on the bootstrap validated Cox model. The calibration curve (Supplementary Figure S1) illustrates how the predictions from the nomogram compare with actual outcomes. The corresponding information for the nomogram for TTP can be found in Supplementary Figure S2.

### Prognostic index

A simplified prognostic index was constructed for the 5-year OS using seven independent prognostic factors. The index score is based on the sum total of factors, with points given for each of the following: treatment, HPV/p16-status, age, pack years, T-status, N-status, and performance status. Risk is assigned as follows: index score 0 to 4, low (≥ 80% probability of surviving 5 years); index score 5 to 7, intermediate (< 80% and > 20% probability of surviving 5 years); and index score 8 or greater, high risk (≤ 20% probability of surviving 5 years) (Table 2).

**Table 2:** Prognostic index for overall survival

Points	0	1	2	3	
Treatment	RT and chemotherapy	RT	Palliative	NA	No treatment
Age	0–44	45–64	65-89	90 and above	NA
HPV/p16	HPV+/p16+	HPV–/p16+	HPV–/p16– or HPV+/p16–	NA	NA
T classification	T1	T2-T3	T4	NA	NA
N classification	N0	N1-N2	N3	NA	NA
Pack years	0–59	59–179	180 and above		
Performance status	0–1	2–3	NA	NA	4

Footnote: It is possible to obtain between 0 and 19 points. Low-risk is from 0–4 points (≥ 80 % risk of being alive after 5 years), medium risk 5–7 points (between < 80 and > 30% chance of being alive after 5 years), and high-risk > 7 points (≤ 30% change of being alive after 5 years).

## DISCUSSION

This study included the largest-to-date collection of 1,542 OPSCCs from a consecutive, population-based, non-selected cohort and assessed OS, TTP, and SAP as well as their relation to multiple clinico-pathological characteristics. From these results, we constructed and internally validated nomograms to predict OS and TTP. Furthermore, we established a simplified prognostic index based on the independent covariates for OS for use in categorizing patients into low-, intermediate-, and high-risk groups. Besides being useful for interpretation of the underlying Cox model, the nomogram combines independent prognostic factors and considers the importance of each on the probability of survival and progression. The prognostic models we present may facilitate discussions in clinical settings and aid in identifying low-risk patients that could be candidates for de-escalation therapy, as well as high-risk patients eligible for new clinical trials. In particular, HPV+/p16+ patients with T1-T2 tumours should be considered candidates for de-escalation therapy, as we demonstrated their survival is similar to the background population, and this would avoid the morbidity associated with therapy. Notably, at least nine de-escalation treatment trials are on-going or finishing [18]. Our nomograms are likely to be applicable to these and future trials, as we reported similar 5-year survival or progression rates as studies in North America;[19–21] Western [22–24], Southern [25], and Northern Europe [6, 26]; Australia [27]; and China [28].

An estimated 10% of all head and neck SCCs are p16+, but HPV– caused by alternative cellular misconfigurations leading to p16-overexpression.[29, 30] Therefore, it is suboptimal to include patients in de-escalation trials based on evaluation of a single biomarker (i.e. HPV or p16 alone) due to the risk of misclassification of tumours and thereby misallocation of patients with an undesired prognosis. [31, 32] One of the main findings in this study includes the identification of HPV+/p16+ as an important and independent predictor for improved OS, TTP, and SAP. Importantly, even though the subgroup of HPV+/p16+ patients was more likely to have a shorter smoking history and a better performance status, even when adjusted for these covariates, HPV+/p16+ was a significant and strong predictor for OS. The calibration for this model was robust and could explain 79% of the observed variability in OS in the cohort. Our findings are in accordance with similar studies addressing survival and progression in HPV+ or p16+; although, we present the first results of a population-based cohort with long-term follow-up. While another smaller study in a region with low HPV-prevalence (below 20%) constructed nomograms for OS and TTP in OPSCC, this study was not population-based and did not address SAP.[33] Furthermore, this study did not convincingly demonstrate which patients were lost to inclusion and follow-up, resulting in less robust external validity.

Our nomograms support the latest AHR-stage classification proposed by O'sullivan *et al.* [34] and comply with the notion that T1–T2 and N0–N2 show significantly better OS, TTP, and SAP compared with ≥ T3 and ≥ N3 tumours, indicating that down-staging of N-classified OPSCCs is reasonable. Noticeably, our findings, in contrast to O'sullivan *et al*, include both HPV and p16 status and patients with distant disease. Our models encourage further studies to better understand whether all N-classified tumours are eligible for down-staging. Although our findings rely on a population-based cohort and selection-bias is minimized, the nomograms have certain limitations. It would be most appropriate to apply the model as the last step in a clinical setting, and the models are not necessarily applicable for changing patients' decisions not to accept further treatment since we do not know her/his response to therapy. With respect to accuracy, the CIs at the various predicted probabilities of recurrence should be considered if using these nomograms in clinical settings. Although this model is internally validated, it could be strengthened by external validation, e.g. in similar population-based cohorts. Future work might focus on validating our results and incorporating additional prognostic factors including robotic, curative surgery, and specific salvage treatment for relapsed disease as well as further outcome measures such as time to treatment or to pathological evaluation.

## MATERIALS AND METHODS

**Figure 3:**
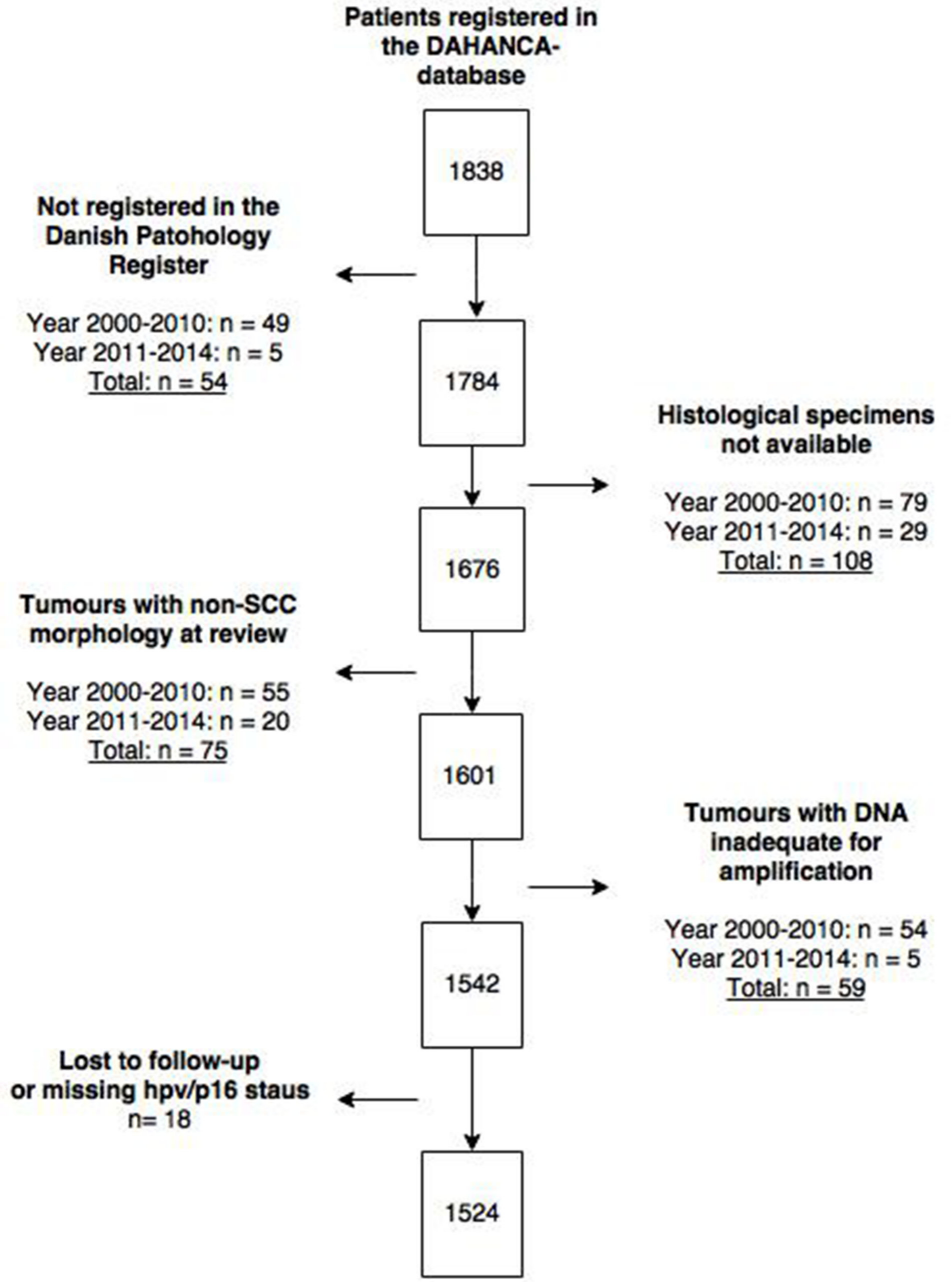
CONSORT flow-diagram

We included patients diagnosed with OPSCC in Eastern Denmark between 2000–2014. The reasons for exclusion are illustrated in Figure 3. We targeted all patients diagnosed with OPSCC in the Eastern part of Denmark cohort with detailed information on tumour (HPV, p16, stage, site) and clinical characteristics (follow-up, treatment, progression). Using the unique resident-code from the Danish Civil Registration System, we linked two national registries, the prospectively maintained Danish Head and Neck Cancer Group (DAHANCA) [35] database and the Danish Pathology Data Registry (DPDR) [36], to identify patients. Patient-data were retrieved from these databases as well as from medical records. The patient cohort from 2000 to 2010 has previously been described [37, 38].

### Tumour-site, histology, tumour-grade, HPV- PCR, and p16 immunohistochemistry

An H&E-stained section of each tumour was reviewed by an expert head and neck pathologist. Based on the finding of tissue-specific structures and clinical information, tumours were divided into base of tongue squamous cell carcinoma (BSCC) and specified- and non-specified tonsillar squamous cell carcinomas (STSCC and NSTSCC). The latter was according to the certainty of origin. The pathologist validated the diagnosis of OPSCC. The p16 staining was considered positive if there was a strong and diffuse nuclear and cytoplasmic reaction in more than 75% of the tumour cells [39]. The HPV DNA-PCR was performed with DNA isolated from FFPE tumour sections. Methods for immunohistochemistry and HPV-PCR have previously been described [37, 38].

### Overall survival, time to progression, and statistics

The date of the last follow-up was the 1st of November 2015. The OS was defined as the time from diagnosis of OPSCC to death due to any cause. Data on vital status (death) stems from the real-time, national, patient-administrative *Green System (GSOpen).* Progression was only considered if it was biopsy-verified. Data on progression stem from the DPDR. The TTP was defined as the time from diagnosis of OPSCC to time of progression at any site. In the analysis of TTP, patients were censored either at the last date of follow-up or at time of death. Patients alive at the last date of follow-up were censored in survival analyses for OS and SAP. The date of diagnosis was used to reflect the date of treatment, since the vast majority of Danish patients initiate treatment within 1 month [40, 41]. The SAP was defined as the time from progression to death due to any cause. Curative radiotherapy regimens consisted of 66–68 GY, divided into 33–34 fractions given 6 days a week. From 2007, stage III-IV patients were offered concomitant cisplatin, if tolerated. Covariates available for adjustment are described in Table 1. The following variables were coded as continuous variables in the survival analyses: age and year of diagnosis. The remaining variables were coded as categorical variables unless otherwise stated. Data on smoking (20 cigarettes per day for 1 year = 1 pack year) were retrieved from the DAHANCA database or medical files. Kaplan-Meier curves were used to illustrate survival differences and significant differences were assessed with log-rank tests. Hazard ratios for death, OS, and TTP were calculated by univariate Cox-regression with log-rank tests for each parameter (Table 1). To evaluate which covariates independently influenced survival, we performed multivariate cox regression analyses with the same factors used in the univariate analyses (Table 3 and Supplementary Tables S2 and S3) with backward elimination, until only significant factors remained. The stopping rule in the backward elimination of factors was based on Akaike's Information Criteria, with the statistics based on the pooled residual chi-square of the model with the R package rms and the function fastbw [42]. The models were internally validated with 200 bootstrappings. The difference in Somers' D between the training and test set was used to evaluate the optimism in the predictive accuracy (i.e. a measure of the difference between observed and predicted values). Somers' D can range from −1 (all pairs disagree) to 1 (all pairs agree) and can be converted to the more well-known AUC via the following formula, AUC = Dyx/2 + 0.5. The calibration of the models was subsequently tested using the rms package in R. To test whether the assumption of proportional hazards was violated, we plotted Schoenfeld residuals for the univariate analyses [43]. Additionally, the multivariate models for survival were examined for violations of the proportional hazards assumption with the function cox.zph. None of the final models violated the proportional hazards assumption. To test for correlations between the covariates, we used Spearman's rank correlation (Figure 1D) [44]. The analysis comparing survival in the cohort with the background population was performed for each year of observation according to the Kaplan-Meier survival rates in the cohort. The survival analysis for the background population was performed for a gender and age-matched population in the same calendar-years as the cohort with mortality figures from the Danish National Department of Statistics (http://www.statistikbanken.dk/statbank5a/default.asp?w=1344). Missing data were left out of the analysis, and variables with missing values are illustrated in Table 1. A *p* value < 0.05 was considered significant. Data were analysed with SPSS version 23 (SPSS Inc., Chicago, IL, USA) and *R* statistics version 3.03.

**Table 3:** Independent covariates for overall survival in the multivariate Cox regression model

	Hazard ratio for death	Lower CI	Upper CI	*P*
**T classification**				
T1	Ref			
T2	1.55	1.13	2.11	0.0060
T3	2.32	1.69	3.19	< 0.0001
T3	3.59	2.52	5.11	< 0.0001
**N classification**				
N0	Ref			
N1	1.59	1.17	2.17	0.0034
N2	2.00	1.54	2.60	< 0.0001
N3	3.59	2.48	5.19	< 0.0001
**Treatment**				
Radiotherapy	Ref			
Chemoradiotherapy	0.7	0.6	0.9	0.0042
Palliative	2.4	1.7	3.5	< 0.0001
No treatment	7.2	4.2	12.1	< 0.0001
**HPV/p16**				
HPV+/p16+	Ref			
HPV+/p16–	2.5	1.8	3.7	< 0.0001
HPV–/p16+	2.1	1.4	3.0	0.0001
HPV–/p16–	3.4	2.7	4.3	< 0.0001
**Age**	1.03	1.01	1.04	< 0.0001
**Pack years**	1.005	1.002	1.008	0.0015
**Performance score**				
0	Ref			
1	1.3	1.1	1.7	0.0105
2	2.1	1.5	2.9	< 0.0001
3	1.6	0.9	2.8	0.0905
4	7.9	2.1	29.9	0.0024

Footnote: CI, 95% confidence interval. The hazard ratio for age and pack years represents the increase in hazard ratio per 1 year increase.

### Validation and calibration of multivariate cox regression models

When performing multivariate analysis of survival, it is of interest to examine how well the models can explain the variation in survival observed in the cohort. The Somers' D for the final model for OS was 0.60, which corresponds to an AUC of 0.80. This was validated with bootstrapping to prevent possible over fitting using the Somers' D rank correlation for the predicted log hazard and observed survival time. The optimism in the model was 0.0064, and the expected bias-corrected AUC was 0.79, suggesting that the model was not over fitted. Finally, the models were evaluated for calibration accuracy in predicting the probability of surviving 5 years. This was performed with bootstrapping to estimate the optimism from the models for how the predicted 5-year survival estimates compared with the observed survival estimates (Supplementary Figure S1). The corresponding AUC and optimism in the multivariate Cox regression model for TTP were 0.68 and 0.010, respectively, and the calibration curve is shown in Supplementary Figure S2.

## SUPPLEMENTARY MATERIALS FIGURES AND TABLES


