# Supplementary material for: Novel nomograms for survival and progression in HPV+ and HPV- oropharyngeal cancer: a population-based study of 1,542 consecutive patients
Source: Oncotarget. 2016 Sep 29;7(44):71761–72. doi: 10.18632/oncotarget.12335 (PMC5342120; doi:10.18632/oncotarget.12335)
Supplement: Supplementary file 1 [file oncotarget-07-71761-s001.pdf]

Novel nomograms for survival and progression in HPV+ and HPV– oropharyngeal cancer: a population-based study of 1,542 consecutive patients

Supplementary Materials

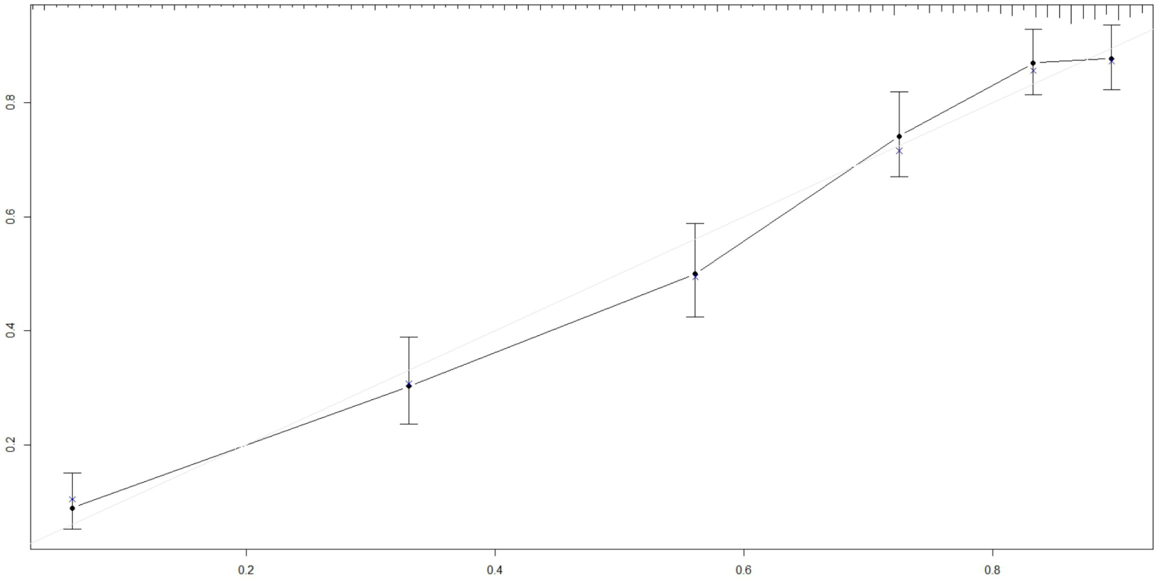

Footnote: Concordance index 0.79. Grey line represents the ideal relationship.

Supplementary Figure S1: Calibration curve for the OS model for surviving 5 years.

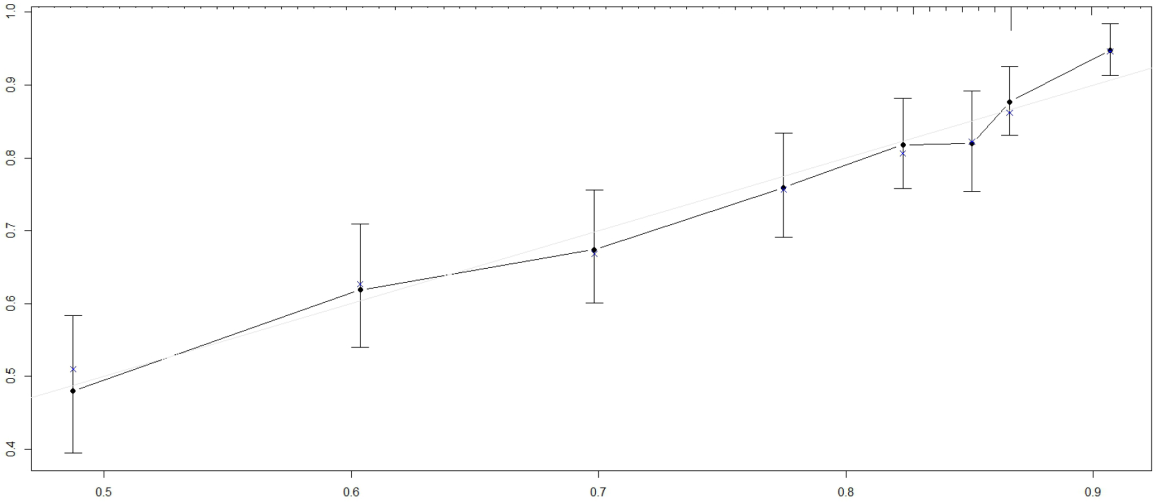

Footnote: concordance index: 0.68. Grey line represents the ideal relationship.

Supplementary Figure S2: Calibration curve for the TTP model for surviving 2 year.

Supplementary Table S1: Independent covariates for overall survival in the multivariate Cox regression model

	Hazard ratio for death	Lower CI	Upper CI	*P*
**T classification**				
T1	Ref			
T2	1.55	1.13	2.11	0.0060
T3	2.32	1.69	3.19	< 0.0001
T3	3.59	2.52	5.11	< 0.0001
**N classification**				
N0	Ref			
N1	1.59	1.17	2.17	0.0034
N2	2.00	1.54	2.60	< 0.0001
N3	3.59	2.48	5.19	< 0.0001
**Treatment**				
Radiotherapy	Ref			
Chemoradiotherapy	0.7	0.6	0.9	0.0042
Palliative	2.4	1.7	3.5	< 0.0001
No treatment	7.2	4.2	12.1	< 0.0001
**HPV/p16**				
HPV+/p16+	Ref			
HPV+/p16–	2.5	1.8	3.7	< 0.0001
HPV–/p16+	2.1	1.4	3.0	0.0001
HPV–/p16–	3.4	2.7	4.3	< 0.0001
**Age**	1.03	1.01	1.04	< 0.0001
**Pack years**	1.005	1.002	1.008	0.0015
**Performance score**				
**0**	Ref			
**1**	1.3	1.1	1.7	0.0105
**2**	2.1	1.5	2.9	< 0.0001
**3**	1.6	0.9	2.8	0.0905
**4**	7.9	2.1	29.9	0.0024

Footnote: CI, 95% confidence interval. The hazard ratio for age and pack years represents the increase in hazard ratio per 1 year increase.

Supplementary Table S2: Patient characteristics and their relationship to time to progression.

Covariate	n	95% CI
**Gender**	1542	
Women	424	(ref)
Male*	1118	1.4 (CI: 1.1-1.8)
**Age**	1542	0.995 (CI: 0.98-1.005)
**Year of diagnosis***	1542	0.97 (CI: 0.95-0.996)
**Pk years, cont.****	1395	1.011 (CI: 1.008-1.014)
**Pk years, cat.**	1395	
0 pk-years	380	(ref)
1-10 pk years	104	1.1 (CI: 0.66-1.9)
11-20 pk years	135	1.4 (CI: 1.0-2.0)
>20 pk years*	776	2.6 (CI:2.0-3.5)
**Tumor location**	1542	
NSTSCC	355	(ref)
STSCC**	764	0.38 (CI: 0.32-0.45)
BOT**	423	0.75 (CI: 0.58-0.98)
**HPV/p16 Status**	1502	
HPV+/p16+	826	(ref)
HPV+/p16-**	76	2.0 (CI: 1.7-3.8)
HPV-/p16+**	93	2.0 (CI: 1.4-3.0)
HPV-/p16-**	507	3.0 (CI: 2.0-3.7)
**Treatment**	1468	
RT	751	(ref)
RT and chemotherapy	585	0.8 (CI: 0.67-1.0)
Palliative*	96	1.8 (CI: 1.1-2.8)
No treatment**	36	2.8 (CI: 1.3-5.8)
**T-stage**	1489	
T1	297	(ref)
T2**	654	1.5 (CI: 1.1-2.0)
T3**	361	2.7 (CI: 2.0-3.8)
T4**	177	3.5 (CI: 2.4-5.0)
**N-stage**	1493	
N0	299	(ref)
N1	271	1.1 (CI: 0.79-1.6)
N2*	800	1.3 (CI: 1.0-1.8)
N3**	123	2.0 (CI: 1.3-2.9))
**M-stage**	1488	
M0	1455	(ref)
M1**	33	4.4 (CI: 2.5-7.6)
**TNM-stage**	1491	
Stage I	48	(ref)
Stage II	150	2.4 (CI: 0.90-5.1)
Stage III	312	2.0 (CI: 0.89-4.7)
Stage IV*	981	2.9 (CI: 1.3-6.6)
**Center**	1501	
Copenhagen	814	(ref)
Herlev	687	0.89 (CI: 0.73-1.1)
**Performance score**	1157	
0	772	(ref)
1*	283	2.9 (CI: 2.4-3.5)
2	71	6.5 (CI: 4.9-3.5)
3	27	0.42 (CI: 0.14-1.3)
4	4	0.05 (CI: 0.00-105)

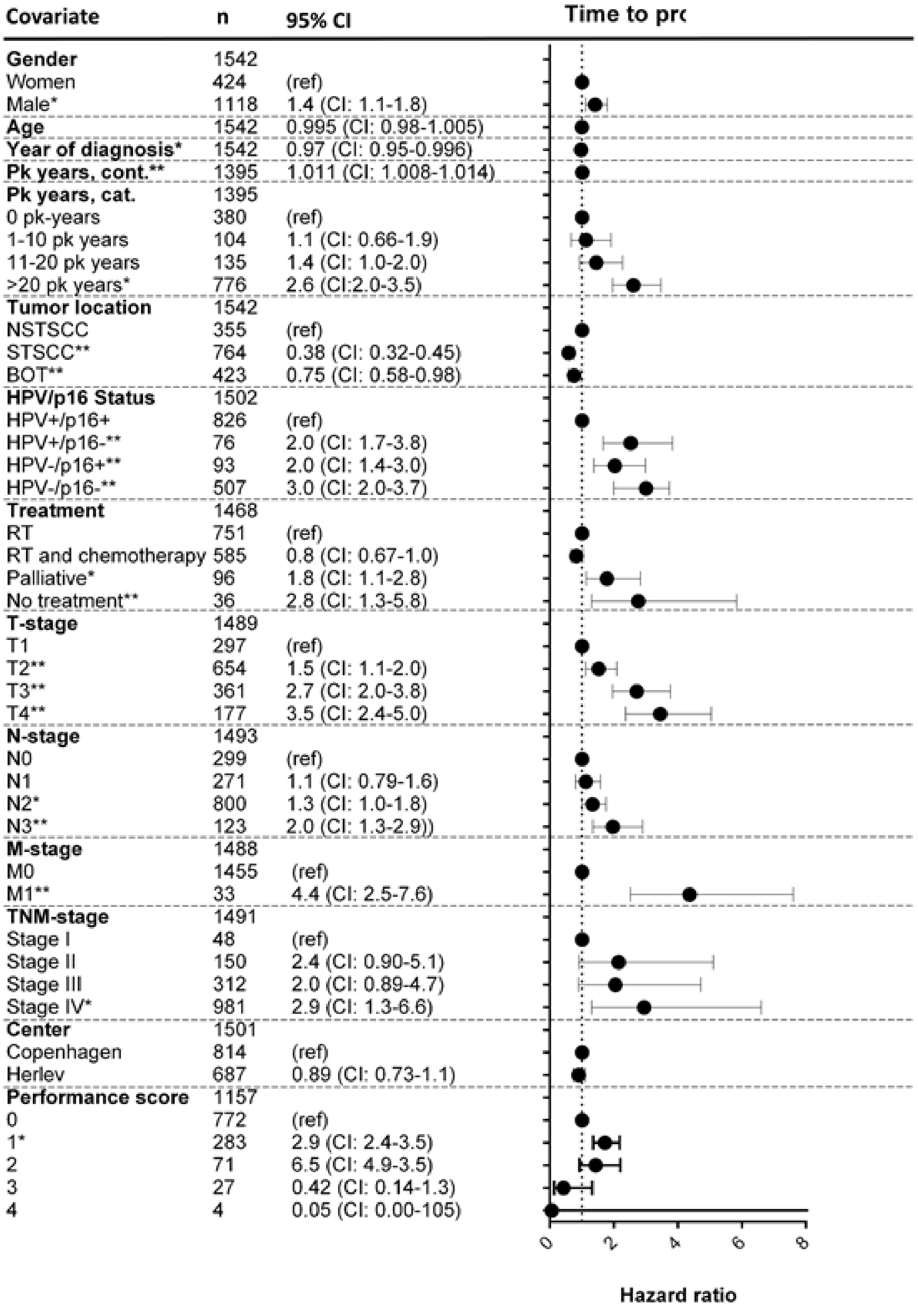

Footnote: The hazard ratios for age, year of diagnosis, and pack years represents the hazard ratio per year increase. RT: radiotherapy. BSCC: Base of tongue squamous cell carcinoma. STSCC and NSTSCC: Specified- and non-specified tonsillar squamous cell carcinomas.

Supplementary Table S3: Independent covariates for time to progression in the multivariate Cox regression model

	Hazard ratio for progression	Lower CI	Upper CI	*P*
**N classification**				
N0	Ref			
N1	1.4	1.0	2.0	0.0580
N2	1.9	1.4	2.6	< 0.0001
N3	2.5	1.6	3.8	< 0.0001
**HPV/p16**				
HPV+/p16+	Ref			
HPV+/p16−	2.3	1.5	3.6	0.0001
HPV-/p16+	1.8	1.2	2.7	0.0042
HPV-/p16−	3.0	2.3	3.8	< 0.0001
**Pack years**	1.008	1.004	1.011	< 0.0001

Footnote: CI, 95% confidence interval. The hazard ratio for pack years is per 1 pack year increment.

Supplementary Table S4: Independent covariates for survival after progression in multivariate Cox regression model

	Hazard ratio for death	Lower CI	Upper CI	*P*
**Progression location**				
Local	Ref			
Regional	0.5	0.4	0.8	0.0005
Distant	1.3	1.0	1.8	0.0424
**HPV/p16**				
HPV+/p16+	Ref			
HPV+/p16−	1.9	1.2	3.2	0.0101
HPV–/p16+	1.9	1.2	3.0	0.0069
HPV–/p16−	2.2	1.6	2.9	< 0.0001
**Performance score**				
**0**	Ref			
**1**	1.4	1.1	1.9	0.0127
**2**	2.3	1.4	3.6	0.0005
**3**	7.6	2.3	24.5	0.0007

Footnote: CI, confidence interval.
